# Analysis of a Skating Time-Trial Competition and Associated Performance-Determinants in Cross-Country Skiers

**DOI:** 10.3390/ijerph191811580

**Published:** 2022-09-14

**Authors:** Lei Shang, Øyvind Sandbakk, Ruiying Shi, Xiaoping Chen, Rune Kjøsen Talsnes

**Affiliations:** 1 Institute of Competitive Sports, Beijing Sport University, Beijing 100084, China; 2 Centre for Sports Research, China Institute of Sport Science, Beijing 100061, China; 3 Centre for Elite Sports Research, Department of Neuromedicine and Movement Science, Norwegian University of Science and Technology, 7030 Trondheim, Norway; 4 National Academy of Economic Strategy, Chinese Academy of Social Sciences, Beijing 100006, China; 5 Meråker High School, Trøndelag County Council, 7735 Steinkjer, Norway or rune.k.talsnes.nord.no; 6 Department of Sports Science and Physical Education, Nord University, 8026 Bodø, Norway

**Keywords:** endurance sport, global positioning system, pacing, inertial measurement unit, peak oxygen uptake, sub-technique selection, XC skiing

## Abstract

**Purpose**: To examine the contributions of time in different terrains and sub-technique distribution to overall time-trial performance, as well as the relationships of laboratory and field-based performance determinants in cross-country skiers. **Methods**: Fourteen male XC skiers were monitored during a 10 km (3 × 3.3 km) skating time-trial competition. On separate days, the skiers performed body composition assessments, laboratory tests while roller-ski skating and a 3 km uphill skating field test. **Results**: Time in uphill terrain was most strongly correlated with overall performance (r = 0.99, *p* < 0.01). G2 and G3 were the predominant sub-techniques (61% of overall time) with more use of G2 on lap three compared to lap one (*p* < 0.05). Body mass and lean mass were inversely correlated with overall and uphill performance (r = −0.60–0.75, all *p* < 0.05). VO_2_ at 4 mmol·L^−1^, VO_2peak_ and TTE while roller-ski skating in the laboratory and the 3 km uphill skating field test correlated with overall performance (r = −0.66–0.85, all *p* < 0.05). **Conclusions**: Time in uphill terrain was the main contributor to overall performance, and G3 and G2 the most used sub-techniques with increased utilization of G2 throughout the competition. VO_2peak_ and TTE while roller-ski skating in the laboratory and performance in an uphill skating field test had the strongest associations with time-trial performance.

## 1. Introduction

Cross-country (XC) skiing is a winter endurance sport with competitions held in hilly terrain with utilization of multiple sub-techniques within the two main techniques, classical and skating [1,2]. The racecourses were designed with approximately one third uphill, one third flat, and one third downhill terrain, leading to ~50% of the overall time being spent in the uphill sections, and ~35% and ~15% in the flat and downhill terrain sections, respectively [1,3,4]. Here, previous studies have shown that work intensity is highest in the uphill sections with performance in this terrain being most important to overall performance in classical and skating time-trials both in distance [3,4,5,6] and sprint competitions [7,8]. Moreover, skiers commonly reduce their speeds throughout time-trial competitions (i.e., positive pacing strategy) [3,9,10,11], although higher ranked skiers are better able to maintain speed compared to lower-ranked skiers [9].

In XC skiing, each terrain section typically lasts between 10 and 90 s [1,10], in which the skiers must effectively select and change between various sub-techniques at different speed and incline combinations [1]. In recent years, the development of combined global navigation satellite systems (GNSS) and inertial measurement units (IMU) has demonstrated a potential to provide concurrent performance analyses of speed and sub-technique selection during training and competitions [12,13,14]. Although the distribution of sub-techniques has previously been extensively investigated in the classical technique [12,14,15,16,17], there is currently limited research into the skating technique [18]. Moreover, skiing speeds are maintained by optimal adjustments of cycle rate (CR) and cycle length (CL) within each sub-technique [2], where previous studies have shown CL to be an important determinant of classical time-trial performance [16]. However, whether the same kinematical patterns and performance determinants apply to skating time-trials requires further elucidation.

XC skiing requires a large aerobic energy contribution, with 70–95% of the total energy expenditure during competitions derived from aerobic energy sources [2], although the terrain-dependent speed and intensity fluctuations elicit an interaction between aerobic and anaerobic energy systems [1,2]. Accordingly, the maximal oxygen uptake (VO_2max_), lactate threshold (i.e., fractional utilization of VO_2max_), and gross efficiency (GE) are key performance determinants in XC skiing [2]. Laboratory-based testing of skiers is commonly used to monitor training-induced changes and to predict performance based on test batteries typically including performance indicators such as VO_2max_ and/or peak oxygen uptakes (VO_2peak_) and threshold-derived measures [11]. In this context, recent studies have demonstrated significant associations between different laboratory tests and performance in a classical time-trial [8], a sprint skating time-trial [8], and a roller-ski skating time-trial [11]. However, laboratory-based performance-determining factors have not yet been associated with an actual on-snow skating time-trial competition. Moreover, inconsistent findings are observed in the above-mentioned studies, likely explained by variations in heterogeneity, statistical power, and performance levels across study samples [11]. This emphasizes a need for better understanding the role of laboratory-based performance determinants in XC skiing. 

In addition to laboratory testing, field-based tests are commonly applied in XC skiing. While such tests are easy to perform and may have high ecological validity, they are associated with higher influence of external factors and lower test-retest reliability [11]. In a recent study by Talsnes et al. [11], both an uphill running and roller-ski double-poling time-trial revealed moderate to large correlations with XC skiing performance. However, the significance of on-snow field-based tests to XC skiing performance has not yet been investigated.

Therefore, the purpose of the present study was to examine the contribution of time in different terrain sections and sub-technique distribution to the overall performance in a 10 km skating time-trial competition, as well as the relationships to laboratory and field-based performance determinants in XC skiers.

## 2. Methods

### 2.1. Participants

Fourteen male skiers (12 classified as Tier 3: highly trained/national level and 2 as Tier 4: elite/international level [19]) were asked to participate in the study. All skiers were a part of the Chinese national team during the 2021–2022 season. Participant characteristics, including International Ski Federation (FIS) distance points are shown in Table 1. All skiers were healthy and free of injuries at the time of the study. The study was approved by the Ethics Committee of China Institute of Sport Science and performed in accordance with the Declaration of Helsinki. Prior to the study, the skiers provided written informed consent to voluntarily take part and were informed that they could withdraw from the study at any time without providing a reason for doing so.

#### 2.1.1. Overall Design

The skiers performed a 10 km skating time-trial competition while being tracked by a combined GNNS and IMU sensor device in mid-December 2021.The skating time-trial competition was used to assess overall performance and performance in uphill, flat, and downhill terrain sections, as well as pacing strategies, sub-technique selection (Gear2-7), and corresponding kinematical patterns. Within a 3-week period prior to the skating time-trial competition, all skiers completed laboratory and field-based testing. All tests were performed approximately at the same time of day (±2 h) to reduce the influence of circadian variations. The tests consisted of: (1) body composition assessments using dual-energy X-ray absorptiometry (iDXA), (2) a submaximal blood lactate profile and incremental test to exhaustion while roller-ski skating in the laboratory, and (3) a 3 km on-snow uphill skating field test.

#### 2.1.2. 10 km Skating Time-Trial Competition

All skiers performed 40 min of warm up before the time-trial competition. The warm-up protocol consisted of 10 min dynamic stretching followed by 25 min of low-intensity skiing (70–82% of peak heart rate [HR_peak_]) and 5 min moderate-intensity skiing (82–87% of HR_peak_). The skiers’ equipment, including skis, skiing boots, and poles was selected based on the skiers’ individual preferences. The skis were waxed according to the prevailing snow conditions by the national team’s professional waxers before the competition. 

**Figure 1:**
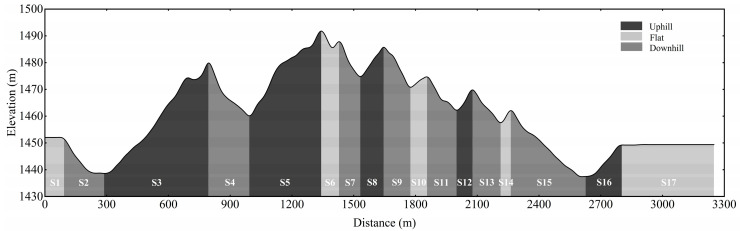
Course and elevation profile of the 3.3 km racecourse used in the skating time-trial competition, including different sections of terrain.

The skating time-trial competition was performed in Xinjiang, China, 1650 m above sea level (m.a.s.l.). The weather conditions were stable during the competition, with an ambient temperature of −3.0 to −5.0 °C and 29.6% humidity. The racecourse consisted of hard-packed snow and was machine groomed the same morning as the competition. The skiers started with a 30 s starting interval and wore a timing chip (Yuandong Future, Beijing, China) on their ankle to record accurate finishing times. Moreover, the skiers’ position, speed, and sub-technique selection during the 10 km skating time-trial were assessed using the Archinisis GNSS system (Archinisis GmbH, Düdingen, Switzerland) including a combined GNNS and IMU sensor. The GNSS data were recorded at 10 Hz and the IMU data at 200 Hz sampling frequency. The 10 km skating time-trial competition consisted of three laps of a 3.3 km racecourse. The racecourse was divided into uphill, flat, and downhill terrain sections, (Figure 1) according to the FIS homologation manual for XC skiing racecourses [20]. Each lap included five uphill sections (S3, S5, S8, S12 and S16) with mean inclines of 4.8°, 5.1°, 4.9°, 6.2° and 4.2°, respectively, seven downhill sections (S2, S4, S7, S9, S11, S13 and S15) with mean inclines of 5.1°, 5.8°, 5.6°, 6.4°, 4.8°, 5.3°, and 4.3°, respectively, and five flat sections (S1, S6, S10, S14 and S17). Detailed kinematical analyses according to Andersson et al. [7] were performed for S3 and S5 (the two major uphill sections) and S17 (the last flat section). 

Archinisis’ algorithm was used for the sub-technique classification. According to the manufacturer, the algorithm was composed of the following steps: (1) the IMU data was fused with the GNSS and barometric pressure data, to obtain acceleration, speed, position, and orientation in the Earth’s global frame; (2) a simplified body model to compute the same values for the approximate center of mass (CoM) (approximately at the level of the belly button); (3) cycle detection based on patterns in the angular speed and in the trunk inclination; (4) sub-technique classification based on a set of features computed for each individual cycle. The sub-techniques were classified using the “gear system”, gear 2-7 (G2-7) as previously described by Andersson et al. [7]. However, G6 (turning technique) was not classified by the current algorithm and constituted ~1–2% of the overall time. During the data collection, the IMU and GNSS sensor device was turned on ~20 min prior to the start to ensure connection with satellites. CoM accuracy and precision were found to be 0.08 m and 0.04 m, respectively. CoM speed accuracy and precision was 0.04 m·s^−1^ and 0.14 m s^−1^, respectively. Ninety-eight per cent of the total time during the 10 km time-trial was correctly classified, and misclassifications predominantly occurred during sub-technique transitions [21,22,23].

### 2.2. Laboratory-Based Tests

#### 2.2.1. Body Composition Assessments

Body composition assessments were performed using the iDXA (General Electric Company, Boston, MA, USA). All skiers were instructed to withdraw from food intake and training the last 8 h before testing. The skiers were further asked to remove all metal items being worn and only wear light clothing. During the test, the skiers were asked to hold their legs together, with palms facing down, the body relaxed, the eyes closed, and to maintain this posture until the end of the test [24]. The iDXA assessed total mass, fat mass, lean mass, and bone mass for the whole body and different body segments.

#### 2.2.2. Blood Lactate Profile and Incremental Test to Exhaustion during Roller-Ski Skating

Prior to the test, the skiers completed a standardized warm up including 10 min running on a treadmill (70–82% of HR_peak_) followed by 10 push-ups and 10 counter-movement jumps. Thereafter, the skiers performed a blood lactate profile in the G3 sub-technique using protocols previously described by Talsnes et al. [25]. The test was performed with a fixed treadmill speed of 2.5 m·s^−1^ and initial incline of 1°, using 5 min stages with increasing incline (workload) of 1° per stage. The rest periods between each stage were 1 min. Respiratory variables and heart rate (HR) were assessed during each stage, whereas rating of perceived exertion (RPE) and blood lactate concentrations (Bla) were determined immediately after each stage. The test was considered complete when the skiers reached a Bla ≥ 4 mmol·L^−1^. Following a 5 min rest period, an incremental test to exhaustion was performed to determine performance indicators (time to exhaustion [TTE]) and VO_2peak_. The test was performed at a constant 6° incline and initial speed of 2.5 m·s^−1^, with a subsequent 0.28 m·s^−1^ increase in speed every minute until exhaustion. VO_2peak_ was defined as the highest VO_2_ averaged over 1 min and HR_peak_ defined as the highest 5 s measurement during the test. RPE and Bla were assessed approximately 1 min after completing the test. HR_peak_ was further applied in the HR calculations of the 10 km skating time-trial competition. 

The same skating roller skis (IDT sports, Lena, Norway) with a weight of 1.92 kg were used by all skiers. Roller skiing was performed on a 3.5 × 2.5 m motor-driven treadmill (RL 3500E, Rodby, Södertalje, Sweden). Respiratory variables were recorded using a breath-by-breath cardiopulmonary system (MetaMax 3B, Cortex Biophysik GmbH, Germany), whereas HR was measured with an ECG-based Polar H7 Bluetooth chest belt (Polar, Kempele, Finland) connected to the MetaMax system. Bla was determined by taking 10 μL of blood from each skier’s fingertip and measured using the Biosen C-Line lactate analyzer (EKF industrial electronics, Magdeburg, Germany). RPE was measured using the 6–20 Borg scale [26].

Work rate was calculated as the sum of power against gravity (P_g_) and power against rolling friction (P_f_) according to Sandbakk et al. [27]. The metabolic rate was calculated as the product of VO_2_ and the oxygen energetic equivalent using the associated respiratory exchange ratio and standard conversion tables. Thereafter, GE was defined as the ratio of work and metabolic rate and [27]. GE was calculated at the same external workload for all skiers (stage 3 during the blood lactate profile). Power output and VO_2_ (fractional utilization) at 4 mmol·L^−1^ Bla was calculated using linear interpolation [28].

### 2.3. Field-Based Test

**Figure 2:**
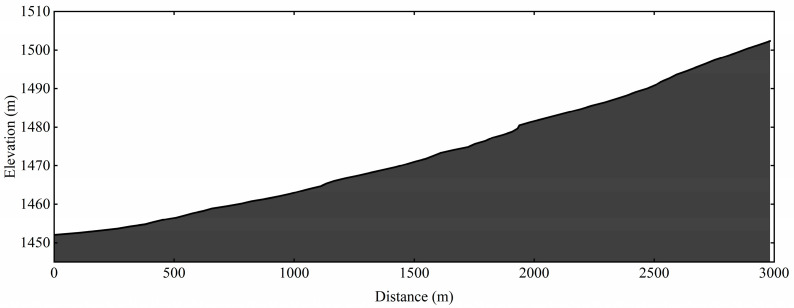
Course and elevation profile of the 3 km uphill skating field test.

The on-snow uphill skating test was performed on a 3 km racecourse with 50.3 m elevation (Figure 2). The test was performed in Xinjiang, China, 1650 m.a.s.l. The weather conditions were stable during the test with ambient temperature of −6.2 °C. The skiers followed similar warm-up procedures as described above for the 10 km skating time-trial. The skiers started with 30 s starting interval and wore a timing chip (Yuandong Future, Beijing, China) on their ankle to record the time. 

### 2.4. Statistical Analyses

Descriptive statistics are presented as mean ± standard deviation for continuous variables. The Shapiro–Wilk procedure was used to test whether the continuous variables met the criteria for normal distribution. A one-way ANOVA was used to compare time, speed, distribution of sub-techniques, and kinetical patterns between laps. The parametric Pearson’s- or non-parametric Spearman’s correlation (RPE and Bla) were used to determine associations between 10 km skating time-trial performance and the laboratory- and field-based performance determinants. Furthermore, stepwise linear regression with overall time as dependent variable and the time spent uphill, flat, and downhill as independent variables was applied. The strength of the correlations was interpreted according to Hopkins et al. [29]: r < 0.1 = trivial, 0.1–0.3 = small, 0.3–0.5 = moderate, 0.5–0.7 = large, 0.7–0.9 = very large, 0.9 = nearly perfect, and 1.0 = perfect. Statistical analyses were performed using IBM SPSS Statistics version 26 software (IBM Analytics, Armonk, NY, USA). The significance levels were set to alpha = 0.05.

## 3. Results

### 3.1. 10 km Skating Time-Trial Competition

Overall time in the skating time-trial competition was 22:20 ± 0:53 min (Table 2). The competition revealed a significant correlation with distance FIS points (r = 0.83, *p* < 0.01), and was therefore a valid measure of the skiers’ performance level. Time in uphill, downhill, and flat terrain constituted 61.4 ± 0.7%, 28.0 ± 0.7%, and 10.6 ± 0.2% of the overall time, respectively. Time spent in uphill, flat, and downhill terrains all significantly correlated with the overall time (*p* < 0.01, Table 2). With respect to each terrain section, all sections displayed significant correlations with the overall time (all *p* < 0.01, Table 2) except for S2 and S4 (downhill terrain). Stepwise multiple regression analyses demonstrated that time in uphill, flat, and downhill terrains explained 97.5% (R^2^ change = 0.975, std. β = 0.77), 0.2% (R^2^ change = 0.002, std. β = 0.14), and 2.3% (R^2^ change = 0.023, std. β = 0.14) of the total variance in overall time-trial performance, respectively (all independent variables: *p* < 0.01). 

**Figure 3:**
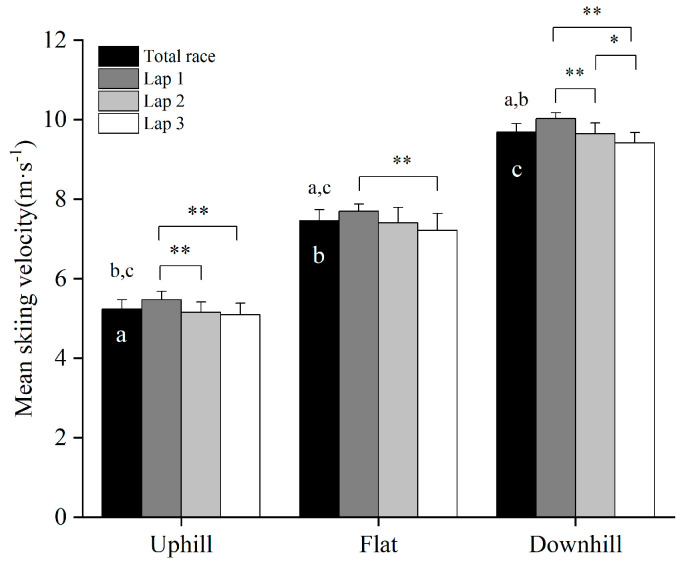
Average velocity in different terrains for each of the three laps and the entire 10 km skating time-trial competition in fourteen national-level male cross-country skiers. * Significant difference between laps (*p* < 0.05); ** Significant difference between laps (*p* < 0.01); ^a^ significantly different from uphill; ^b^ significantly different from flat; ^c^ significantly different from downhill (all, *p* < 0.05).

The one-way ANOVA showed a significant difference in the average speed between laps (F = 14.71, *p* < 0.01, Table 2) with an 8.6 ± 3.1% reduction in speed from lap one to lap three (*p* < 0.01). The speed reduction from lap one to lap three was distributed as 7.0 ± 3.5%, 6.3 ± 4.1%, 6.1 ± 1.5% in the uphill, flat, and downhill terrain, respectively (all *p* < 0.05, Figure 3). The skiers’ average and highest obtained HR during the time-trial competition were 173 ± 7 beats·min^−1^ and 181 ± 6 beats·min^−1^, respectively, corresponding to 91 ± 2% and 96 ± 1% of HR_peak_. After finishing the time-trial competition, the skiers demonstrated a peak Bla of 14.3 ± 2.9 mmol·L^−1^.

**Figure 4:**
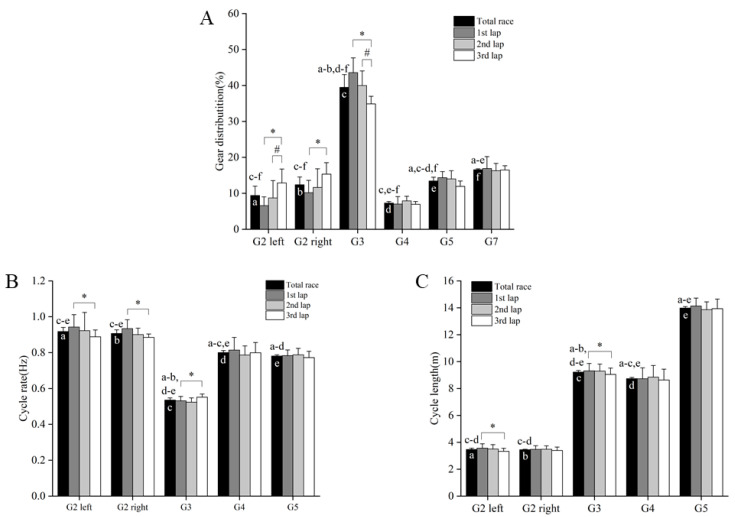
(**A**) Gear distribution, (**B**) cycle rate, and (**C**) cycle length for each lap and the entire 10 km skating time-trial competition in fourteen national-level male cross-country skiers. * Significant difference between lap1 and lap3; ^#^ Significant difference between lap2 and lap3 (*p* < 0.05); ^a^ Significantly different from G2 left; ^b^ Significantly different from G2 right; ^c^ Significantly different from G3; ^d^ Significantly different from G4; ^e^ Significantly different from G5; ^f^ Significantly different from G7 (all *p* < 0.05).

During the time-trial competition, the total number of movement cycles was 777 ± 40, with a mean cycle rate and cycle length of 0.79 ± 0.05 Hz and 6.23 ± 0.56 m, respectively (excluding the G5-G7 sub-technique). Six different sub-techniques were utilized during the competition (Figure 4a), with G2 and G3 being the predominant sub-techniques constituting 61.2 ± 2.3% of the overall time. The relative distribution of sub-techniques together with mean cycle rates and cycle lengths are displayed in Figure 4b,c. The skiers performed 4.6 ± 6.2% and 8.8 ± 7.0% more movement cycles during lap two and lap three compared to lap one (*p* < 0.05). Moreover, there was a significant increase in the use of G2 (left side) from lap two to lap three (4.2 ± 3.1%-point, *p* < 0.05), and G2 (both sides) from lap 1 to lap 3 (6.3 ± 3.8%-point and 5.2 ± 4.5%-point for G2 left and G2 right, respectively, *p* < 0.05). These changes were accompanied by a significant reduction in the use of G3 from lap two to lap three, and from lap one to lap three (5.1 ± 3.5%-point and 8.7 ± 3.4%-point, respectively, *p* < 0.05). Within each sub technique, there was a significant reduction in CR for G2 (both sides) from lap one to lap three (0.05 ± 0.04 Hz and 0.05 ± 0.04 Hz for G2 left and G2 right, respectively, *p* < 0.05), and a 0.22 ± 0.19 m significant reduction in CL from lap one to lap three for G2 left (*p* < 0.05). Lastly, there was a significant 0.02 ± 0.01 Hz increase in CR and 0.26 ± 0.23 m reduction in CL for G3 from lap one to lap three (*p* < 0.05). 

Kinematical changes between laps for G2 or G3 in the two major uphill sections (S3 and S5) and the last flat section (S17) are shown in Table 3. These findings were consistent with the abovementioned changes found on a lap-to-lap basis, demonstrating a significant reduction in cycle rate within both G2 and G3 from lap one to lap two, and from lap one to lap three in S3 (*p* < 0.05). These findings were accompanied with a significant reduction in CL within G2 from lap one to lap two, and from lap one to lap three (*p* < 0.05, Table 3). 

### 3.2. Laboratory- and Field-Based Determinants of Performance

Descriptive data from the body composition assessments and their correlation with the time-trial competition are presented in Table 4. Total body mass and total lean mass were significantly correlated with overall time and time in all terrains of the time-trial competition (all, *p* < 0.05). Similar correlations were found between lean mass in the different body segments and overall time and time in uphill terrain (−0.75–0.74, all *p* < 0.05). In addition, lean mass in the arms was significantly correlated with time in flat and downhill terrain (−0.67 and −0.66, both *p* < 0.05) of the time-trial competition. 

**Figure 5:**
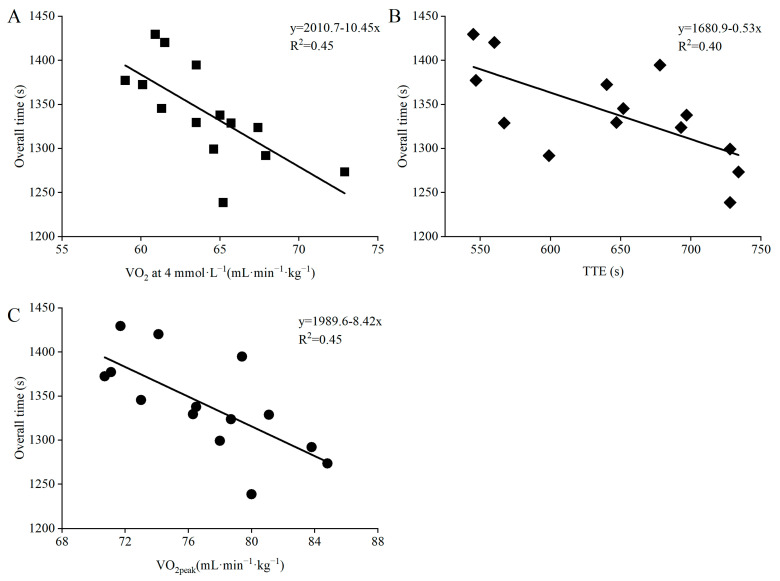
Relationship between 10 km skating time-trial performance and (**A**) oxygen uptake at of 4 mmol·L^−1^ blood lactate, (**B**) peak oxygen uptake (VO_2peak_), and (**C**) time to exhaustion (TTE) in roller skating during laboratory testing in fourteen national-level male cross-country skiers. Presented with individual data points and trend lines based on linear regression.

Descriptive data of the laboratory-based performance determinants are presented in Table 5 and their correlation with the time-trial competition in Table 6 and Figure 5. During submaximal roller skiing, oxygen uptake (VO_2_) at 4 mmol·L^−1^ Bla was significantly correlated with overall time, time per lap and time in all terrains of the time-trial competition (all *p* < 0.01). Moreover, power at 4 mmol·L^−1^ Bla was significantly correlated with overall time, time on lap two, and time in uphill and downhill terrain (all *p* < 0.05). During incremental roller skiing to exhaustion, VO_2peak_ was significantly correlated with overall time and time spent in all terrains of the time-trial competition (Table 6, both *p* < 0.05). TTE only displayed significant correlations with overall time, time on lap three, as well as time in uphill and downhill terrain (*p* < 0.01). The mean time in the field-based uphill skating test was 07:51 ± 00:18 min and was significantly correlated with overall time, time on each lap, and time in all terrains of the time-trial competition (all *p* < 0.05, Table 6). 

## 4. Discussion

The present study examined the contribution of time in different terrain sections and sub-technique distributions to overall performance in a 10 km skating time-trial competition, as well as the relationships to laboratory and field-based performance determinants in XC skiers. The main findings were as follows: (1) time in uphill terrain was the main contributor to overall performance, (2) the skiers adopted a positive pacing strategy with reduced speeds in all terrains throughout the competition, (3) G2 and G3 were the predominant sub-techniques constituting 62% of the overall time, with increased utilization of G2 throughout the competition, (4) higher total and lean body mass were associated with better performances, and (5) out of the performance-determining factors, VO_2_ and power at 4 mmol·L^−1^ Bla, VO_2peak_, and TTE during roller-ski skating in the laboratory, as well as performance in a 3 km on-snow uphill skating field test, had the strongest associations with time-trial performance.

### 4.1. Analyses of the Skating Time-Trial Competition

The nearly perfect correlation found between time in uphill terrain and overall time-trial performance expands upon previous research demonstrating similar associations both in distance- [3,4,5,6] and sprint time-trials in XC skiing [7,8]. Nearly perfect and very large correlations between time in flat and downhill terrains and the overall performance were also found, but the stepwise multiple regression analyses showed that most of the variance was shared with the contribution from uphill performance. In fact, only 2% of the remaining variance in the stepwise regression was explained by time in flat and downhill terrains, demonstrating high multi-collinearity between the independent variables. Altogether, these findings emphasize a significant contribution of all terrain types with the best performing skiers being generally faster in all terrains. However, uphill-specific performance was clearly the main determinant of 10 km skating time-trial performance in a group of national-level male skiers. 

The skiers in the present study adopted a positive pacing strategy with a ~9% speed reduction from lap one to lap three. These findings are consistent with previous research indicating the use of a positive pacing strategy during time-trial competitions in XC skiing [3,9,10,11], although it has been demonstrated that higher-ranked skiers demonstrate a more even pacing compared to lower-ranked skiers [9]. The observed speed reductions were distributed across all terrain types but most of the reductions in speed from lap one to lap three occurred in the uphill terrain sections, in line with previous findings during a classical time-trial competition among female skiers [4]. However, the speed reductions found in the present study were somewhat higher than the typically 2–4% decreases in speed seen over the second half of time-trials among skiers [9]. This may in part be explained by differences in racecourses and/or the competition altitudes (e.g., ~1650 m.a.s.l in the present study) which may have increased the demands for a more even pacing strategy [30]. Alternatively, this may be related to differences in pacing expertise between study samples. Interestingly, a recent intervention by Losnegard et al. [31] demonstrated that skiers with a fast-start pattern improved skating time-trial performance by adopting a slower start and thus more even pacing. Therefore, considering the relatively large speed reductions found in the present study, it is likely that these skiers would have benefited from adopting a more even pacing strategy. 

### 4.2. Analyses of Sub-Technique Selection and Kinematical Patterns

The G2 and G3 were the most predominant sub-techniques during the skating time-trial competition, constituting 20% and 40% of the overall time, respectively. Moreover, only 7% was G4, whereas 10% and 16% of the overall time were spent in the G5 (skating without poles) and G7 (tuck position) sub-techniques, respectively. These findings are comparable with the sub-technique distribution found during a sprint skating time-trial [7], although the minor (~1–2%) distribution of G6 (turning technique) was not included in the present study due to classification errors. Interestingly, the 16% time spent in the G7 (tuck position) in the present study was higher than previously found during a sprint skating time-trial [7] and in classical time-trial competitions [15,16]. These differences are likely explained by variations in the degree of ascent and descent between different racecourses. However, considering that ~61% of the overall time was spent in uphill terrain where the largest performance differences occurred, developing the G2 and G3 sub-techniques (62% of the overall time) should be prioritized in the training process of skiers to improve skating time-trial performance. 

Coinciding with the reduced speeds throughout the time-trial competition, the skiers reduced their utilization of G3 and increased the utilization of the G2 sub-technique from lap two to lap three, and particularly from lap one to lap three. The speed reductions and corresponding changes in sub-technique selection further coincided with reductions in CL within G2 and G3 from lap one to lap three. These findings agree with those found in the double-poling technique during a 10 km classical time-trial competition, demonstrating reduced CL throughout the competition [16], as well as more laboratory-based findings, emphasizing CL as an important performance determinant in the skating technique [1,2]. Overall, speed reductions seem to be related to the use of “lower gears” and reduced ability to generate propulsion and CL, indicating that the use of “higher gears” and long CL are important determinants of skating time-trial performance and are also associated with a more even pacing strategy. 

### 4.3. Laboratory- and Field-Based Performance Determinants

The moderate to large inverse relationship between total mass, lean mass, and time-trial performance indicated that the heaviest skiers with most lean mass were the fastest skiers in both overall time and time in different terrains. These findings are somewhat in conflict with previous studies demonstrating no correlations between body mass and body composition measures and XC skiing performance [7,11]. However, comparable associations have been found between lean mass and performance among both female [32] and male skiers [33], and particularly in sprint XC skiing [34,35]. The reason for the present findings may be related to the advantages of having better upper- and lower-body strength for propulsion among the skiers with most lean mass, which is supported by the same associations found between lean mass in the arms and performance in flat and downhill terrain. This is further supported by Larsson and Henriksson–Larsèn suggesting the importance of lean body mass in the arms to XC skiing performance in junior male skiers [33]. Another explanation may be the fact that some of the lower performing skiers in the present study had a background in running, and thus a lower body and lean mass before transferring to XC skiing in a talent transfer initiative as previously described by Talsnes et al. [25]. 

The laboratory-derived performance indicator (TTE), as well as both absolute and body-mass normalized VO_2peak_ while roller-ski skating, demonstrated moderate to very large correlations with overall time and time in all terrains of the time-trial competition. These findings agree with previous studies, emphasizing the importance of a high aerobic energy turnover in XC skiing [2,4,11]. Moreover, both VO_2peak_ and TTE displayed a larger correlation with time on lap three compared to lap one, in which the latter is in line with a previous study investigating 10 km biathlon sprint competition [36]. This indicates that better performing skiers use different pacing strategies than their lower performing counterparts, and that incremental testing while roller-ski skating in the laboratory is relevant for determining performance at the end of an on-snow skating time-trial competition. The large to very large correlations found between VO_2_ at 4 mmol·L^−1^ Bla and time-trial performance probably reflect the higher VO_2peak_ values found among the better performing skiers, as no significant correlations were found between fractional utilization (%VO_2peak_) and the time-trial performance. The higher VO_2_ at 4 mmol·L^−1^ Bla probably also explains the moderate to large correlations found between power output at 4 mmol·L^−1^ Bla and time-trial performance. Overall, the present findings emphasize the validity of laboratory-derived performance and physiological measures in XC skiing, which can be used to determine performance and monitor training progress. However, the test-retest reliability and actual ability to detect training-induced changes across time using such tests should be examined further. 

The 3 km on-snow uphill skating test demonstrated large to very large correlations with overall time and time in all terrains of the time-trial competition. These findings are consistent with a recent study demonstrating significant correlations between uphill field tests in both running and double poling with XC skiing performance in a group of national-level male skiers [11]. The large correlations found between the field test and the time-trial performance are likely explained by the fact that both tests were performed on snow in the same area, and under the same external conditions. This was further strengthened by the largest correlations found between the uphill skating test and performance in uphill terrain and lap three of the time-trial competition. Although associated with higher influence of external factors and lower test-retest reliability (e.g., wind, temperature, and snow friction) than standardized laboratory-tests, the present study demonstrates that performance in an uphill skating field test showed very large associations with performance and provides an easy-to-use practically relevant field test. 

### 4.4. Methodological Considerations

Whilst care was taken to ensure scientific quality, the present study included some limitations. First, there was a 3 week gap between the competition and the other tests. However, the participants were highly trained elite athletes, had a stable performance level during this period and similar training regimes and preparation before all tests. Second, the participants’ motivation to perform such extensive testing and differences in skis and equipment may have influenced the results, although the latter was minimized by using the same ski wax and professional waxer for all participants. Third, correlation analyses only provide information about the validity of different laboratory- and field-based performance tests. Hence, the test-retest reliability and actual ability to detect training-induced changes across training cycles using such tests should be examined further.

## 5. Conclusions

The present findings reveal that time spent in uphill terrain is the main contributor to overall performance in skating time-trial competitions among national-level male skiers. While G3 and G2 were the predominant sub-techniques utilized, a positive pacing pattern (i.e., reduced speeds) throughout the competition was followed by increased utilization of G2 coinciding with reduced cycle lengths. From the laboratory-derived values, a high lean body mass together with high VO_2peak_ and TTE during roller-ski skating were highly associated with performance. However, performance in an uphill skating field test also showed very large associations with performance and provides an easy-to-use practically relevant field test. Altogether, these findings emphasize that high aerobic energy turnover to perform well in the uphill sections, as well as the corresponding sub-techniques with sufficiently long cycle lengths, should be development-areas for improving skating time-trial performance in male skiers. 

## Figures and Tables

**Table 1:** Participant characteristics of the fourteen national-level male cross-country skiers participating in the study.

Age (years)	22.6 ± 3.4
Body height (cm)	177.7 ± 5.5
Body mass (kg)	69.4 ± 5.2
Body mass index (kg·m^−2^)	22.0 ± 2.1
VO_2peak_ (mL·min^−1^·kg^−1^)	77.1 ± 4.4
^a^ Weekly training volume (h)	22.4 ± 0.5
Distance FIS points	78.4 ± 21.7

Data are presented as mean ± standard deviation. VO_2peak_, peak oxygen uptake in roller-ski skating; FIS, International Ski Federation. ^a^ Average weekly training volume during the annual training cycle.

**Table 2:** Distance, time, and speed for overall distance, laps, and terrain sections, as well as correlations with overall time during a 10 km skating time-trial in fourteen national-level male cross-country skiers.

	Distance (m)	Time (s)	Speed (m·s^−1^)	Correlation (r)
1. lap	2775.9 ± 4.8	424.8 ± 13.4	6.54 ± 0.20	0.88 **
2. lap	2772.4 ± 4.8	450.4 ± 19.9	6.17 ± 0.26	0.89 **
3. lap	2774.2 ± 3.7	465.1 ± 24.7	5.98 ± 0.32	0.95 **
Overall	8322.5 ± 8.8	1340.2 ± 52.8	6.22 ± 0.25	1.00
S3 (uphill)	497.3 ± 0.5	123.3 ± 7.0	4.07 ± 0.22	0.90 **
S5 (uphill)	348.6 ± 1.0	85.7 ± 4.3	4.09 ± 0.20	0.99 **
S8 (uphill)	115.5 ± 0.5	18.3 ± 0.8	6.33 ± 0.27	0.80 **
S12 (uphill)	73.2 ± 0.8	11.7 ± 0.6	6.28 ± 0.28	0.83 **
S16 (uphill)	191.6 ± 0.3	35.5 ± 2.1	5.42 ± 0.32	0.88 **
Overall uphill	1226.3 ± 3.1	823.5 ± 40.7	5.24 ± 1.00	0.99 **
S6 (flat)	85.9 ± 0.8	35.7 ± 1.4	7.24 ± 0.24	0.71 **
S10 (flat)	83.7 ± 1.3	30.2 ± 1.1	8.35 ± 0.30	0.84 *
S14 (flat)	50.0 ± 1.1	19.1 ± 0.9	7.91 ± 0.27	0.83 **
S17 (flat)	116.9 ± 0.9	56.4 ± 3.8	6.26 ± 0.43	0.91 **
Overall flat	336.7 ± 0.2	141.4 ± 6.8	7.44 ± 0.29	0.91 **
S2 (downhill)	156.9 ± 1.0	20.9 ± 0.4	7.55 ± 0.14	0.53
S4 (downhill)	198.3 ± 0.5	19.1 ± 0.3	10.41 ± 0.16	0.36
S7 (downhill)	100.8 ± 0.8	9.6 ± 0.3	10.55 ± 0.30	0.61 *
S9 (downhill)	125.1 ± 0.5	13.3 ± 0.3	9.44 ± 0.25	0.84 **
S11 (downhill)	142.2 ± 1.1	13.8 ± 0.3	10.34 ± 0.29	0.84 **
S13 (downhill)	140.1 ± 0.8	15.3 ± 0.5	9.17± 0.33	0.76 **
S15 (downhill)	345.5 ± 0.1	33.2 ± 0.8	10.41 ± 0.24	0.88 **
Overall downhill	1208.9 ± 3.3	375.4 ± 7.5	9.69 ± 1.01	0.85 **

Data are presented as mean ± standard deviation. Strengths of the correlations are interpreted as following: r < 0.1 = trivial, 0.1–0.3 = small, 0.3–0.5 = moderate, 0.5–0.7 = large, 0.7–0.9 = very large, 0.9 = nearly perfect, and 1.0 = perfect; * *p* < 0.05; ** *p* < 0.01.

**Table 3:** Speed, distance, and kinematics in the G2 and G3 sub-technique in two selected uphill sections (S3 and S5) and the flat section (S17) per lap during a 10 km skating time-trial in fourteen national-level male cross-country skiers.

Lap	Speed (m s^−1^)		Distance (m)		Cycle Rate (Hz)		Cycle Length (m)	
**S3**	**G2**	**G3**	**G2**	**G3**	**G2**	**G3**	**G2**	**G3**
1	3.40 ± 0.22	4.87 ± 0.38	62.2 ± 4.8	461.7 ± 5.1	0.92 ± 0.06	0.56 ± 0.03	3.72 ± 0.23	8.75 ± 0.66
2	3.12 ± 0.15 *	4.50 ± 0.21 *	135.1 ± 4.7 *	388.6 ± 4.5 *	0.88 ± 0.04 *	0.52 ± 0.02 *	3.55 ± 0.23 *	8.61 ± 0.59
3	3.06 ± 0.22 ^#^	4.48 ± 0.25 ^#^	184.1 ± 20.6 ^#^	339.8 ± 20.5 ^#^	0.88 ± 0.05 ^#^	0.51 ± 0.01 ^#^	3.47 ± 0.29 ^#^	8.48 ± 0.57
**S5**								
1	3.10 ± 0.22	4.70 ± 0.17	93.0 ± 2.8	391.5 ± 3.9	0.91 ± 0.04	0.52 ± 0.02	3.43 ± 0.30	9.11 ± 0.48
2	2.89 ± 0.19 *	4.59 ± 0.26	100.3 ± 2.9	380.9 ± 4.2	0.88 ± 0.02 *	0.52 ± 0.03	3.28 ± 0.20 *	8.90 ± 0.52
3	3.02 ± 0.29	4.56 ± 0.31	125.6 ± 5.4 ^#^	328.9 ± 7.2 ^#^	0.92 ± 0.08	0.50 ± 0.02 ^#^	3.31 ± 0.35	8.79 ± 0.60
**S17**								
1	--	6.23 ± 0.29	--	96.5 ± 2.7	--	0.56 ± 0.03	--	11.17 ± 0.98
2	--	6.20 ± 0.44	--	96.4 ± 2.8	--	0.56 ± 0.03	--	11.14 ± 1.03
3	--	6.33 ± 0.68	--	95.2 ± 2.5	--	0.59 ± 0.03	--	10.68 ± 1.36

Data are presented as mean ± standard deviation. * Significant difference between lap 1 and lap 2 (*p* < 0.05). ^#^ Significant difference between lap 1 and lap 3 (*p* < 0.05).

**Table 4:** Body composition presented in absolute values (kg) and relative to total body mass (%BM) for arms, trunk, and legs, as well as correlations with overall time during a 10 km skating time-trial in fourteen national-level male cross-country skiers.

	**Total Body Mass**		**Total Fat Mass**		**Total Lean Mass**		**Total Bone Mass**	
	Kg	%BM	Kg	%BM	Kg	%BM	Kg	%BM
Total	69.4 ± 5.2	NA	6.7 ± 1.5	9.5 ± 2.0	59.7 ± 4.3	85.9 ± 1.8	3.08 ± 0.29	4.4 ± 0.29
Arms	9.0 ± 0.7	12.9 ± 0.4	0.8 ± 0.2	1.2 ± 0.3	8.1 ± 0.8	11.7 ± 0.8	0.47 ± 0.05	NA
Trunk	32.5 ± 2.7	46.8 ± 1.0	2.7 ± 0.8	3.9 ± 1.0	28.8 ± 2.3	41.5 ± 1.1	0.90 ± 0.12	NA
Leg	23.4 ± 1.9	33.7 ± 1.0	2.3 ± 0.6	3.2 ± 0.8	20.0 ± 1.6	28.8 ± 1.6	1.20 ± 0.12	NA
	**Total Body Mass**		**Total Fat Mass**		**Total Lean Mass**		**Total Bone Mass**	
	Kg	%BM	Kg	%BM	Kg	%BM	Kg	%BM
Overall time (s)	−0.70 *	NA	−0.20	−0.01	−0.75 *	−0.01	−0.49	0.09
Uphill Terrain (s)	−0.69 *	NA	−0.19	−0.01	−0.74 *	−0.01	−0.48	0.10
Flat terrain (s)	−0.65 *	NA	−0.28	−0.12	−0.66 *	0.11	−0.47	0.05
Downhill terrain (s)	−0.60 *	NA	−0.09	0.09	−0.67 *	−0.10	−0.42	0.06

Data are presented as mean ± standard deviation. Kg, kilogram; BM, body mass; NA, not available. Strengths of the correlations are interpreted as following: r < 0.1 = trivial, 0.1–0.3 = small, 0.3–0.5 = moderate, 0.5–0.7 = large, 0.7–0.9 = very large, 0.9 = nearly perfect, and 1.0 = perfect. * *p* < 0.05.

**Table 5:** Laboratory-based data from a submaximal blood lactate profile and time to exhaustion test during roller-ski skating in fourteen national-level male cross-country skiers.

**Submaximal Test (4 mmol·L^−1^)**	
VO_2_ (L·min^−1^)	4.47 ± 0.53
VO_2_ (mL·min^−1^·kg^−1^)	64.2 ± 3.5
HR (bpm)	162.9 ± 7.0
HR in %HR_peak_	86.0 ± 2.1
RPE (6–20)	15.4 ± 0.6
Power (watt)	199.7 ± 27.1
VO_2_ in % VO_2peak_	83.3 ± 1.8
GE (%)	15.9 ± 2.3
**TTE Test**	
VO_2peak_ (L·min^−1^)	5.37 ± 0.64
VO_2peak_ (mL·min^−1^·kg^−1^)	77.1 ± 4.4
TTE (s)	643.9 ± 67.0
RPE (6–20)	19.1 ± 0.5
Bla (mmol·L^−1^)	12.1 ± 2.1
HRp_eak_ (bpm)	189.3 ± 6.6

Data are presented as mean ± standard deviation. VO_2_, oxygen uptake; HR, heart rate; HR_peak_, peak heart rate; RPE, rating of perceived exertion; VO_2peak_, peak oxygen uptake; GE, gross efficiency; Bla, blood lactate concentration.

**Table 6:** Correlations of laboratory and field-based tests with overall performance and performance in different terrains during a 10 km skating time-trial in fourteen national-level male cross-country skiers.

	10-km Skating Time-Trial						
	Overall Time (s)	Lap 1 (s)	Lap 2 (s)	Lap 3 (s)	Uphill Terrain (s)	Flat Terrain (s)	Downhill Terrain (s)
**Laboratory based**							
**Submaximal test (4 mmol·L^−1^)**							
VO_2_ (L·min^−1^)	−0.76 **	−0.72 **	−0.66 **	−0.70 **	−0.74 **	−0.70 **	−0.70 **
VO_2_ (mL·min^−1^·kg^−1^)	−0.67 **	−0.62 **	−0.50	−0.70 **	−0.64 **	−0.62 *	−0.68 **
HR in %HR_peak_	−0.20	−0.02	0.04	−0.45	−0.14	−0.25	−0.39
RPE (6–20)	0.03	−0.18	0.08	0.10	−0.01	0.01	0.20
Power (watt)	−0.62 *	−0.50	−0.68 *	−0.51	−0.61 *	−0.48	−0.60 *
VO_2_ in % VO_2peak_	0.03	−0.18	0.15	0.04	0.12	−0.15	−0.30
GE (%)	0.03	−0.18	0.15	0.04	0.12	−0.15	−0.30
**TTE test**							
VO_2peak_ (L·min^−1^)	−0.76 **	−0.69 **	−0.68 **	−0.70 **	−0.75 **	−0.67 **	−0.64 **
VO_2peak_ (mL·min^−1^·kg^−1^)	−0.65 *	−0.52	−0.53	−0.68 **	−0.66 *	−0.54 *	−0.53
TTE (s)	−0.61 *	−0.52	−0.46	−0.62 *	−0.66 *	−0.42	−0.55 *
RPE (6–20)	0.19	0.10	0.22	0.16	0.23	0.05	0.02
Bla (mmol·L^−1^)	0.20	0.08	0.23	0.19	0.19	0.15	0.20
HR_peak_ (beats·min^−1^)	−0.32	−0.33	−0.14	−0.39	−0.29	−0.39	−0.32
**Field based**							
3 km uphill skating TT(s)	0.88 **	0.85 **	0.71 **	0.86 **	0.86 **	0.82 **	0.80 **

VO_2_, oxygen uptake; HR, heart rate; HR_peak_, peak heart rate; RPE, rating of perceived exertion; VO_2peak_, peak oxygen uptake; GE, gross efficiency; Bla, blood lactate concentration; TT, time-trial. Strengths of the correlations are interpreted as the following: r < 0.1 = trivial, 0.1–0.3 = small, 0.3–0.5 = moderate, 0.5–0.7 = large, 0.7–0.9 = very large, 0.9 = nearly perfect, and 1.0 = perfect. * *p* < 0.05; ** *p* < 0.01.

## Data Availability

Not applicable.
